# Computed-Tomography Body Composition Analysis Complements Pre-Operative Nutrition Screening in Colorectal Cancer Patients on an Enhanced Recovery after Surgery Pathway

**DOI:** 10.3390/nu12123745

**Published:** 2020-12-05

**Authors:** Pamela Klassen, Vickie Baracos, Leah Gramlich, Gregg Nelson, Vera Mazurak, Lisa Martin

**Affiliations:** 1 Department of Agricultural, Food & Nutritional Sciences, University of Alberta, Edmonton, AB T6G2P5, Canada; pamela2@ualberta.ca; 2 Department of Oncology, University of Alberta, Edmonton, AB T6G2P5, Canada; vickie.baracos@ualberta.ca (V.B.); ls2@ualberta.ca (L.M.); 3 Department of Medicine, University of Alberta, Edmonton, AB T6G2P5, Canada; lg3@ualberta.ca; 4 Department of Oncology, University of Calgary, Calgary, AB T6G2P5, Canada; Gregg.Nelson@albertahealthservices.ca

**Keywords:** malnutrition, sarcopenia, colorectal cancer, CT, PG-SGA, subjective global assessment, myosteatosis, muscle mass

## Abstract

Pre-operative nutrition screening is recommended to identify cancer patients at risk of malnutrition, which is associated with poor outcomes. Low muscle mass (sarcopenia) and lipid infiltration to muscle cells (myosteatosis) are similarly associated with poor outcomes but are not routinely screened for. We investigated the prevalence of sarcopenia and myosteatosis across the nutrition screening triage categories of the Patient-Generated Subjective Global Assessment Short Form (PG-SGA_SF_) in a pre-operative colorectal cancer (CRC) cohort. Data were prospectively collected from patients scheduled for surgery at two sites in Edmonton, Canada. PG-SGA_SF_ scores ≥ 4 identified patients at risk for malnutrition; sarcopenia and myosteatosis were identified using computed-tomography (CT) analysis. Patients (*n* = 176) with a mean age of 63.8 ± 12.0 years, 52.3% male, 90.3% with stage I–III disease were included. Overall, 25.2% had PG-SGA_SF_ score ≥ 4. Sarcopenia alone, myosteatosis alone or both were identified in 14.0%, 27.3%, and 6.4% of patients, respectively. Sarcopenia and/or myosteatosis were identified in 43.4% of those with PG-SGA_SF_ score < 4 and in 58.5% of those with score ≥ 4. Overall, 32.9% of the cohort had sarcopenia and/or myosteatosis with PG-SGA_SF_ score < 4. CT-defined sarcopenia and myosteatosis are prevalent in pre-operative CRC patients, regardless of the presence of traditional nutrition risk factors (weight loss, problems eating); therefore, CT image analysis effectively adds value to nutrition screening by identifying patients with other risk factors for poor outcomes.

## 1. Introduction

Globally, colorectal cancer is among the most frequently diagnosed cancers accounting for 1.8 million new diagnoses and 800,000 deaths in 2018 [1]. Surgical resection typically occurs shortly after diagnosis especially in organ-confined disease. Depending on risk factors, resection may be followed by adjuvant chemotherapy to reduce recurrence risk. In locally advanced disease, surgical resection may be preceded by neo-adjuvant chemotherapy and/or radiation and subsequently followed by adjuvant chemotherapy. In these cases, total curative treatment time can be up to 18 months. 

In both surgical and oncological contexts, routine nutrition screening is recommended to identify patients with or at risk of malnutrition, characterized in cancer patients by negative energy balance and skeletal muscle loss [2,3,4]. Malnutrition is associated with longer length of post-operative hospital stay [5], surgical complications [6,7,8,9], and reduced overall survival for cancer patients [10,11,12], therefore early identification of risk factors that can lead to malnutrition (e.g., weight loss, problems eating, poor appetite) is essential. Pre-operative nutritional care, starting with nutrition screening, is one of the tenets of the evidence-based, multi-modal Enhanced Recovery After Surgery (ERAS) protocol. Designed to reduce peri-operative stress, maintain physiological function post-operatively, and promote faster recovery, the widely-accepted protocol consists of approximately 20 recommendations including several related to optimal nutrition care from pre-operative nutrition screening to post-operative early feeding and immuno-nutrition [3]. However, despite general agreement on the benefit of nutrition screening in both surgical and oncological settings, no single screening tool is recommended [2,3,4]. The Patient-Generated Subjective Global Assessment Short Form (PG-SGA_SF_, © FD Ottery, 2001) is a validated screening tool that is commonly used in ambulatory oncology settings. As an abridged version of the patient and clinician-completed Patient-Generated Subjective Global Assessment, it uses patient-reported recent weight loss, symptoms, and difficulty eating as indicators of risk to quickly identify patients who may benefit from further nutritional assessment and intervention [13,14]. 

While traditional indicators of nutrition risk such as weight loss, low BMI, and low oral intake certainly increase risk of malnutrition and associated poor outcomes, evidence has accumulated that features such as computed tomography (CT)-defined low skeletal muscle mass and fat infiltration to the muscle cells are also strongly associated with negative clinical outcomes such as increased risk of post-operative complications [6,15,16], longer post-operative length of stay [17] and reduced overall survival [10,16,18,19,20,21] in oncology patients. In oncology patients, low skeletal muscle mass associated with poor clinical outcomes is referred to as sarcopenia [22]. Although there are various methods of assessing skeletal muscle, CT analysis of cross-sectional images at the third lumbar (L3) vertebrae is considered the gold standard in the oncological setting to precisely quantify skeletal muscle. Not only are CT images routinely available for the majority of patients, this assessment carries no additional patient burden and can be completed prior to an in-person assessment [23]. Other methods of identifying muscle loss do exist, including the full PG-SGA (clinician-completed), which includes a nutrition-focused physical exam [24]. However, sarcopenia can be masked by overweight and obesity and therefore go undetected on physical exams or nutrition risk screening [25,26]. Similarly, sarcopenia-specific screening tools such as SARC-F can be used to identify patients who have functional changes as a result of low muscle mass [27], but does not quantify the muscle. Both tools require additional patient time, contact and clinic space, and neither tool allows for accurate visualization of the muscle. 

With the prolific use of CT body composition analysis in oncology research, low muscle radiodensity (known as myosteatosis) has emerged alongside sarcopenia as an additional prognostic factor. Skeletal muscle radiodensity (SMR, reported in Hounsfield Units, HU) inversely reflects the triglyceride content of skeletal muscle. Low SMR is often referred to as myosteatosis; thresholds for which have been defined according to associations with overall survival after chemotherapy [11] as well as length of stay and hospital readmissions after colorectal cancer surgery [17]. Myosteatosis, as a characteristic of muscle, cannot be identified by any method other than CT analysis.

Since nutrition screening is already part of the pre-operative ERAS pathway, and in light of the known impacts of malnutrition, sarcopenia and myosteatosis on surgical and oncological outcomes, it is relevant to explore the prevalence of sarcopenia and myosteatosis across different levels of nutrition screening results. For advanced cancer patients, prior work confirms that sarcopenia and myosteatosis are prevalent across all levels of nutrition risk (low to high) using a variety of nutrition screening tools [11,28]. In early stage cancer patients, the prevalence of sarcopenia and myosteatosis has been described but not been analyzed in tandem with nutrition screening results [17]. Identifying whether high nutrition screening scores consistently co-exist with sarcopenia and myosteatosis in early stage disease will inform the development of care pathways to ensure that all relevant risk factors are identified early. In the present study, we aimed to describe the prevalence of sarcopenia and myosteatosis according to level of nutrition risk as defined by the PG-SGA_SF_ in patients with CRC presenting for elective surgical resection. We hypothesized that there would be a high prevalence of sarcopenia and myosteatosis across all of the nutrition risk triage categories of the PG-SGA_SF_. 

## 2. Materials and Methods 

Data were prospectively collected from consecutive patients ≥18 years old presenting for pre-operative assessment prior to elective surgical resection of a primary CRC. Data collection occurred at two acute care centres in Edmonton, Alberta between 2016 and 2017; both centres had implemented the Enhanced Recovery After Surgery (ERAS) protocol for colorectal surgery. Patients completed a PG-SGA_SF_ at their first pre-operative visit and the forms were scored by a trained researcher. Patient demographics, cancer stage and cancer site were obtained from the Alberta Cancer Registry. Surgical data (e.g., surgery date and procedure) were obtained from the ERAS Interactive Audit System (EIAS), which has been previously described [5]. Finally, CT images were obtained from the regional Picture Archiving Communication System. Patients were eligible for inclusion if they had completed a pre-operative PG-SGA_SF_, had a confirmed diagnosis and cancer stage, had an analyzable CT image within 6 months prior to surgery, and had complete surgical data available in EIAS. Ethical approval was granted from the local health research ethics board (protocol identifier: HREBA.CC-16-0308).

The PG-SGA_SF_ consists of scored patient-reported components including weight change, changes in food intake, symptoms impacting the ability to eat, and performance status; these scores are summed for a total possible score out of 37 (a higher score represents greater nutrition risk). A nutrition triage recommendation is assigned based on the total PG-SGA_SF_ score as follows: no intervention required (scores 0–1), education or pharmaceutical intervention (scores 2–3), registered dietitian intervention (scores 4–8), or critical nutrient intervention and improved symptom management (scores ≥ 9). For the purposes of this analysis, patients were divided by triage recommendation into two groups—scores 0–3 and scores ≥ 4, with the latter group considered to be at risk for malnutrition.

Body composition was analyzed using CT image analysis, previously validated for use in the cancer population [23]. CT body composition analysis makes opportunistic use of existing CT images, taken in this case as part of the normal staging process for patients with colorectal tumors. Cross-sectional skeletal muscle and adipose tissue areas from a single axial image at the third lumbar vertebrae (L3) are highly correlated with total body skeletal muscle and adipose tissue [29]. L3 images were analyzed using an auto-segmentation module (ABACS module, Voroni Health Analytics; Slice-O-Matic©, Tomovision, Montreal, QC, Canada) and manually corrected by a trained technician. Muscle and adipose tissue cross-sectional areas were delineated in cm^2^ using pre-defined thresholds; −29 to +150 HU for skeletal muscle, −150 to −50 HU for visceral adipose tissue, and −190 to −30 HU for subcutaneous adipose tissue. The resultant cross-sectional areas were normalized for height and reported as skeletal muscle index (SMI), visceral adipose tissue index (VATI), and subcutaneous adipose tissue index (SATI) in cm^2^/m^2^. The mean SMR for the entire muscle area at L3 was recorded. 

Sarcopenia and myosteatosis were defined using previously identified sex- and age-specific SMI and SMR thresholds, which were developed by Martin et al. based on associations with post-operative length of stay in a large cohort of CRC patients undergoing surgery [17]. The cohort of the present study was similar to the cohort of Martin et al., and therefore these thresholds were deemed highly appropriate. The primary outcome of the present study was to determine the prevalence of sarcopenia and myosteatosis across nutrition risk screening categories in pre-operative colorectal cancer patients and therefore was intended as a descriptive study; it was not powered to evaluate the association between these muscle features and surgical outcomes.

Statistical analysis was performed using IBM SPSS Statistics for Windows, version 24 (SPSS, Chicago, IL, USA). Frequency and summary data are presented, with comparisons between groups analyzed using chi square tests with Bonferroni corrections or independent t-tests where appropriate. A *p*-value of <0.05 was considered statistically significant. 

## 3. Results

### 3.1. Patient Characteristics

Completed PG-SGA_SF_, analyzable CT and EIAS data were obtained for 176 patients (Table 1). The sample contained similar proportions of males and females, with a mean age of 63.8 ± 12.0 years. Similar proportions of colon and rectal tumor sites were included, and 90% of patients had stage I–III disease. While data collection aimed to include patients presenting for curative intent surgery, a small proportion were subsequently found to have stage IV disease. Mean BMI was 28.4 ± 6.3 kg/m^2^, with no significant difference between males and females. Overweight (BMI of 25–29.9 kg/m^2^) and obesity (BMI ≥ 30 kg/m^2^) were prevalent, with 42% and 30% of the cohort in these categories, respectively.

### 3.2. Nutrition Risk Factors by Patient-Generated Subjective Global Assessment Short Form

Results from the PG-SGA_SF_ are presented in Table 2. On average, patients presented with minimal weight change over the past month (mean −0.4% ± 3.4%); however, there was wide variability, ranging from 30% weight loss to 13% weight gain. The PG-SGA_SF_ scores the severity of weight loss according to five categories, and our patients with 1 month weight change reported were categorized as follows: 0–1.9% weight loss, 129 (83.2%); 2–2.9% weight loss, 10 (6.5%); 3–4.9% weight loss, 9 (5.8%); 5–9.9% weight loss, 5 (3.2%); and ≥10% weight loss, 2 (1.3%). 

Food intake was unchanged for 84.7% of patients, and 85.2% reported no problems eating. The most frequent nutrition impact symptoms included diarrhea (10.2%), fatigue (9.1%), no appetite (8.0%) and constipation (8.0%). Finally, the vast majority of patients reported normal or fairly normal activity. 

Overall, the mean total PG-SGA_SF_ score was 2.9 ± 4.8 (Table 3), out of a total possible score of 37; scores ranged from 0 to 26. Patients with scores of 0-1 comprised 59.8% of the cohort, indicating no intervention required, and 25.2% of patients scored ≥ 4, indicating a need for dietitian assessment or intervention. Among patients who scored ≥ 4, mean total score was 9.2 ± 5.8.

### 3.3. CT-Defined Skeletal Muscle Analysis

**Figure 1:**
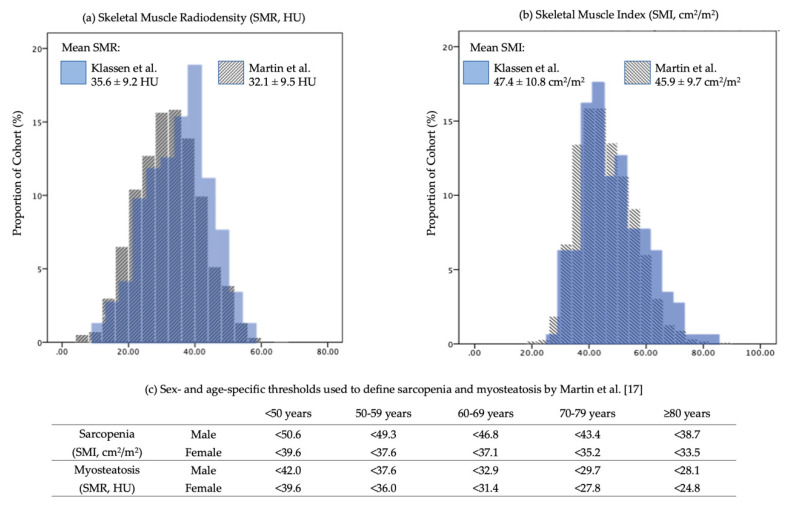
(**a**) and (**b**). Similar distributions of (**a**) skeletal muscle radiodensity and (**b**) skeletal muscle index from two pre-operative colorectal cancer cohorts: the present cohort (*N* = 176) and Martin et al. (*N* = 2100) [17]. (**c**). Martin et al. thresholds for sarcopenia and myosteatosis using skeletal muscle index (SMI) and skeletal muscle radiodensity (SMR), respectively, developed based on associations with longer post-operative length of stay in early-stage colorectal cancer patients [17].

Mean SMI for males and females were 53.3 ± 9.8 and 40.9 ± 7.7 cm^2^/m^2^, respectively, with a mean of 47.4 ± 10.8 cm^2^/m^2^ overall. SMR for males and females was 35.5 ± 9.1 and 35.7 ± 9.3 HU, respectively, with an overall mean of 35.6 ± 9.2 HU. The distributions of these features were consistent with the analysis of a large cohort (*N* = 2100), similar CRC cohort described by Martin et al., shown in Figure 1a and b. Based on the thresholds developed by Martin et al. [17] (Figure 1c), sarcopenia alone was identified in 14.0%, myosteatosis alone in 27.3%, and the combination of both in an additional 6.4% (Table 1) in our cohort. More females than males presented with sarcopenia alone (19.8% vs. 8.8%, *p* < 0.05), and more males than females presented with combined sarcopenia and myosteatosis (9.9% vs. 2.5%; *p* < 0.05). However, there was no difference between sexes in the overall prevalence of sarcopenia and/or myosteatosis (49.5% of males vs. 46.3% of females). 

### 3.4. Co-Existence of Nutrition Risk by PG-SGA_SF_ and CT-Defined Sarcopenia and Myosteatosis

**Figure 2:**
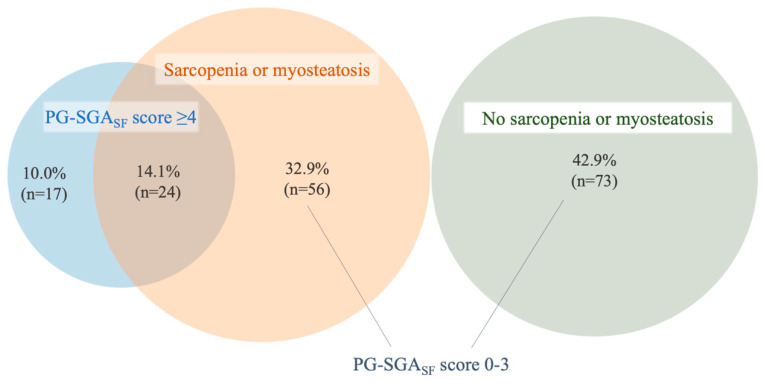
Proportional Venn diagram illustrating the co-existence of PG-SGA_SF_ score ≥ 4 and sarcopenia or myosteatosis pre-operatively. Sarcopenia or myosteatosis co-existed with a PG-SGA_SF_ score of 0–3 in 32.9% of the total cohort.

The prevalence of sarcopenia and myosteatosis according to PG-SGA_SF_ triage category was evaluated. Sarcopenia and/or myosteatosis were prevalent both above and below a PG-SGA_SF_ cutoff score of ≥4. Of the patients with scores < 4 (75.9% of the cohort), 43.4% had CT-defined sarcopenia and/or myosteatosis, compared to 58.5% of those with screening scores ≥ 4, with no significant difference between groups, as shown in Table 4. Of the entire cohort, only 14.1% had co-existing PG-SGA_SF_ score ≥ 4 and sarcopenia or myosteatosis. One-third (32.9%) had sarcopenia or myosteatosis with PG-SGA_SF_ score < 4 (Figure 2), and therefore were identified by nutrition screening as having low risk of malnutrition but were found to have sarcopenia or myosteatosis using CT analysis.

## 4. Discussion

### 4.1. Early Nutrition Risk Exists 

In this pre-operative, CRC cohort treated with curative intent, one-quarter of patients presented with PG-SGA_SF_ scores ≥ 4 early in their treatment journey. This cohort represents a demographic that is not well-characterized in the literature with respect to nutrition screening than patients with unresectable or metastatic disease, for whom the prevalence of nutrition risk ranges from 36 to 64% (i.e., PG-SGA_SF_ score ≥ 4 or SGA B/C) [6,11,13,30]. The lower prevalence of PG-SGA_SF_ score ≥ 4 in our cohort compared to cohorts with advanced disease may reflect a smaller window of time for weight loss and functional decline to occur from disease onset to assessment, and that nutrition impact symptoms are less common prior to chemotherapy treatment. However, it is likely that early malnutrition will progress over the course of treatment due to the stress of surgery and nutrition impact symptoms caused by adjuvant chemotherapy [31]. For these patients, the pre- and peri-operative periods are essential times to intervene and attempt to halt the progression of malnutrition [32]. 

### 4.2. Sarcopenia and Myosteatosis Are Prevalent in Pre-Operative CRC Patients

Sarcopenia and myosteatosis were prevalent across PG-SGA_SF_ triage categories and regardless of sex, with 48% of the cohort having at least one of these muscle features. While sarcopenia and myosteatosis do not necessarily occur together, both are independently associated with poor outcomes [17]. In the short term, pre-operative sarcopenia and myosteatosis carry increased risks of post-operative complications [6,15], longer length of stay and hospital readmission [17]. Patients with sarcopenia at diagnosis who continue to lose muscle over the next two years have significantly worse overall survival than those with stable or increased muscle mass [33]. Our prevalence data suggest that sarcopenia and myosteatosis are less common early in the disease trajectory compared to advanced stage but are still widespread. For example, Ní Bhuachalla et al. analyzed 725 CRC patients, 45% with stage IV disease, and found 41% to have sarcopenia and 46% to have myosteatosis, compared to our results of 20 % and 34%, respectively [28]. Yet, most patients with stage II–III CRC require adjuvant chemotherapy after surgery or future chemotherapy upon recurrence, where sarcopenia and myosteatosis are known to be associated with increased treatment toxicity, poor quality of life and decline in functional status [18,19]. Although nutrition elements exist within the ERAS pathway, nutrition screening alone without body composition analysis remains the primary method of identifying patients requiring nutrition intervention [3]. Early identification of sarcopenia and myosteatosis could enable prompt intervention, prevent progressive muscle degradation, and further optimize outcomes. 

### 4.3. Nutrition Risk and Skeletal Muscle Aberrations Are Distinct Risk Factors

This co-analysis of CT-defined sarcopenia and myosteatosis alongside PG-SGA_SF_ nutrition screening corroborates the work of others showing that low nutrition screening scores do not preclude risk for poor outcomes conferred by sarcopenia and myosteatosis [11,12,28]. Despite three-quarters of our cohort having low PG-SGA_SF_ screening scores (0–3), nearly half of these had pre-existing sarcopenia and/or, myosteatosis. CT analysis, therefore, identified one-third (32.9%) of the total cohort as having sarcopenia and/or myosteatosis early in the treatment journey, in the absence of traditional nutrition risk factors. In light of this, we suggest that patients with muscle aberration are a distinct population, requiring unique screening tools. In early stage disease, sarcopenia and myosteatosis may not be related to reduced oral intake, which the PG-SGA_SF_ would identify, but rather may be pre-existing and related to low baseline activity levels, poor diet quality, or comorbid conditions such as COPD or diabetes, as described by Xiao et al [34]. This recognition does not discount the positive predictive value of nutrition screening, but rather emphasizes the need for complementary screening in early stage cancer to identify patients with sarcopenia and myosteatosis. Furthermore, efforts are required to identify the risk factors predisposing patients to these aberrations, and particularly those which are amendable to intervention. 

### 4.4. Enhancing Identification of At-Risk Patients

Our results confirm that pre-operative CRC patients commonly have sarcopenia or myosteatosis in the absence of reductions in food intake and weight, and therefore may not be selected for early intervention using traditional nutrition screening tools. The pre-operative consultation is an opportune time to assess patients not only for weight loss or change in oral intake, but also for existing sarcopenia and myosteatosis. CT analysis is the most suitable tool in this population, given that 73% of our cohort was overweight or obese, with a significant number having myosteatosis which is otherwise not identified by physical or functional assessments. Furthermore, CT analysis avoids increasing the patient time requirement in the screening process and requires similar or less time from a clinician than an in-person assessment. The limitations of CT analysis should be acknowledged, including the requirement for trained personnel, the cost of for-purpose software, and the requirement for CT images at the L3 vertebrae to be available. These limitations, however, are quickly being addressed. In the oncology context, CT images are routinely available. Furthermore, free versions of the analysis software are now widely used, and efforts are ongoing to validate analysis at other locations such as first lumbar vertebrae and twelfth thoracic vertebrae in populations without abdominal CT scans [35].

**Figure 3:**
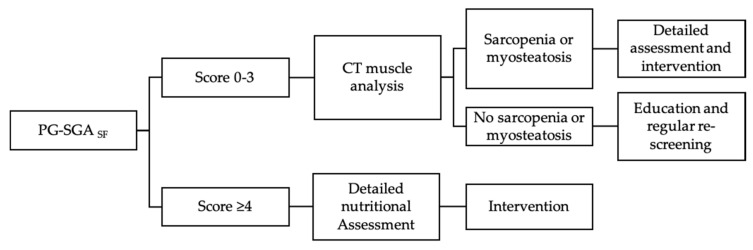
Proposed clinical flow for surgical oncology patients starting with nutrition screening, followed by CT muscle analysis for patients with low screening scores, to identify patients with skeletal muscle aberration in the absence of overt nutrition risk.

Future work in this area should focus on the feasibility of integrating screening for sarcopenia and myosteatosis with nutrition screening processes, thereby accurately identifying both risk factors. A clinical flow for surgical oncology that includes PG-SGA_SF_ screening followed by CT analysis of body composition for patients with low screening scores (Figure 3) would effectively capture patients with traditional malnutrition risk factors and those with sarcopenia or myosteatosis. Further detailed assessment after this screening is warranted, and could include dietary assessment or functional assessments depending on the setting. In non-oncology settings, the use of an alternative screening tool such as SARC-F to complement nutrition screening may similarly be useful to identify sarcopenia [36], despite its inability to detect myosteatosis. In either case, the improved identification of these distinct groups of at-risk patients will set the stage for research into effective interventions, improved clinical care pathways and multi-modal pre-habilitation programs using exercise, nutrition and psychosocial support [37,38,39,40,41] which have yet to be formally included in ERAS programs. Through a combination of enhanced screening and targeted interventions, the pre-operative period can become an opportunity to enhance outcomes from cancer surgery through survivorship. 

## Figures and Tables

**Table 1:** Characteristics of pre-operative colorectal cancer patients.

Demographics	Male	Female	All (*N* = 176)
Age (years), mean (±SD)	63.6 (10.7)	63.9 (13.3)	63.8 (12.0)
Sex, *N* (%)	92 (52.3)	84 (47.7)	
*Tumor site, N (%)*			
colon	45 (48.9)	47 (45.0)	92 (52.3)
rectum	47 (51.1)	37 (44.0)	84 (47.7)
*Cancer stage, N (%)*			
Stage I–II	51 (55.4)	46 (54.7)	97 (55.1)
Stage III	32 (34.8)	30 (35.7)	62 (35.2)
Stage IV	5 (5.4)	5 (6.0)	10 (5.7)
*Anthropometrics*			
Weight, kg, mean (±SD)	90.7 (18.2)	69.9 (17.2)	80.7 (20.5)
Height, cm, mean (±SD)	176.5 (7.0)	159.0 (8.6)	168.3 (11.7)
BMI, kg/m^2^, mean (±SD)	29.0 (5.0)	27.8 (7.5)	28.4 (6.3)
*BMI category, kg/m^2^, N (%)*			
<20	2 (2.2)	9 (11.1)	11 (6.5)
20–24.9	15 (16.9)	21 (25.9)	36 (21.2)
25–29.9	41 (46.1)	31 (38.3)	72 (42.4)
30–34.9	19 (21.3)	10 (12.3)	29 (17.1)
35–39.9	10 (11.2)	4 (4.9)	14 (8.2)
≥40	2 (2.2)	6 (7.4)	8 (4.7)
*Body composition by CT analysis*			
Mean skeletal muscle index (SMI), cm^2^/m^2^	53.3 (9.8)	40.9 (7.7)	47.4 (10.8)
Mean skeletal muscle radiodensity (SMR), HU	35.5 (9.1)	35.7 (9.3)	35.6 (9.2)
Subcutaneous adipose tissue index (SATI, cm^2^/m^2^), mean	67.1 (29.0)	103.6 (58.0)	84.1 (48.4)
Visceral adipose tissue index (VATI, cm^2^/m^2^), mean	79.7 (38.1)	46.9 (36.9)	64.3 (40.9)
Sarcopenia, myosteatosis or both, *N* (%)	45 (49.5)	38 (46.3)	83 (48.0)
*Sarcopenia alone, N (%)*	*8 (8.8)*	16 (19.8) *	*24 (14.0)*
*Myosteatosis alone, N (%)*	*28 (30.8)*	*19 (23.5)*	*47 (27.3)*
Sarcopenia and Myosteatosis, *N* (%)	9 (9.9) *	2 (2.5)	11 (6.4)
No sarcopenia or myosteatosis, *N* (%)	46 (50.5)	44 (53.7)	90 (52.0)

Cancer Stage: American Joint Committee on Cancer 7th Edition; * *p* < 0.05.

**Table 2:** Nutritional risk factors by PG-SGA_SF_.

Domain	Overall, *N* = 176
**Box 1: Weight Change**	
Weight change past month, mean % (±SD)	−0.4 (3.4)
Weight change past 6 months, mean % (±SD)	−2.0 (5.5)
No change/increased weight in past 2 weeks, *N* (%)	123 (69.9)
Decreased weight in past 2 weeks, *N* (%)	53 (30.1)
**Box 2: Food Intake**	
*Food intake past month, N (%)*	
Unchanged/more than usual	149 (84.7)
Less than usual	27 (15.3)
*Type of food intake, N (%)*	
Normal food, normal amount	143 (81.3)
Normal food, less than normal amount	18 (10.2)
Little solid food	3 (1.7)
Only liquids or nutritional supplements	8 (4.5)
Very little of anything	4 (2.3)
Only tube feeding/feeding by vein	0 (0)
**Box 3: Nutrition Impact Symptoms, *N* (%)**	
No problems eating	150 (85.2)
No appetite	14 (8.0)
Nausea	7 (4.0)
Constipation	14 (8.0)
Diarrhea	18 (10.2)
Vomiting	3 (1.7)
Feel full quickly	6 (3.4)
Foods taste funny or have no taste	3 (1.7)
Smells bother me	3 (1.7)
Mouth sores	0 (0)
Problem swallowing	3 (1.7)
Fatigue	16 (9.1)
Pain	4 (2.3)
Dry mouth	7 (4.0)
Other	4 (2.3)
**Box 4: Activity and Function, *N* (%)**	
Normal, no limitations	115 (65.3)
Not normal self, fairly normal activities	44 (25.0)
Not feeling up to most things, in bed or chair <half day	8 (4.5)
Not able to do most things or pretty much bedridden	7 (4.0)

PG-SGA_SF_, Patient-Generated Subjective Global Assessment Short Form.

**Table 3:** Patient-Generated Subjective Global Assessment Short Form scores and triage recommendation.

PG-SGA_SF_ Domain	Score 0–3	Score ≥ 4	Overall
Box 1: Weight Change (max. 5; mean ± SD)	0.27 (0.64)	1.77 (1.38)	0.64 (1.10)
Box 2: Food Intake (max. 5; mean ± SD)	0.13 (0.53)	1.68 (1.74)	0.52 (1.19)
Box 3: Nutrition Impact Symptoms (max. 24; mean ± SD)	0.08 (0.41)	4.59 (4.30)	1.20 (2.92)
Box 4: Activity and Function (max. 3; mean ± SD)	0.22 (0.50)	1.18 (0.95)	0.47 (0.77)
Total Score, mean ± SD	0.70 (1.05)	9.23 (5.77)	2.86 (4.79)
**Triage Recommendation**			*N*, %
0–1 (no intervention, reassess regularly)			104 (59.8)
2–3 (patient/family education; pharmacological intervention as indicated by symptoms)			26 (14.9)
4–8 (intervention by RD and nurse or physician as indicated by symptoms)			26 (14.9)
≥9 (critical need for symptom management and nutrition intervention)			18 (10.3)

RD, registered dietitian; max., maximum score possible.

**Table 4:** Prevalence of sarcopenia and myosteatosis by PG-SGA_SF_ score.

Characteristic	PG-SGA_SF_ Score 0–3	PG-SGA_SF_ Score ≥ 4
Sarcopenia alone, *N* (%)	15 (11.6)	7 (17.1)
Myosteatosis alone, *N* (%)	30 (23.3)	17 (41.5)
Sarcopenia and myosteatosis, *N* (%)	11 (8.5)	0
